# Author Correction: Structural basis of Nipah virus RNA synthesis

**DOI:** 10.1038/s41467-025-58404-2

**Published:** 2025-03-27

**Authors:** Fernanda A. Sala, Katja Ditter, Olexandr Dybkov, Henning Urlaub, Hauke S. Hillen

**Affiliations:** 1 Department of Cellular Biochemistry, University Medical Center Göttingen, Göttingen, Germany; 2 Research Group Structure and Function of Molecular Machines, Max Planck Institute for Multidisciplinary Sciences, Göttingen, Germany; 3 Bioanalytical Mass Spectrometry Group, Max Planck Institute for Multidisciplinary Sciences, Göttingen, Germany; 4 Bioanalytics Group, Institute for Clinical Chemistry, University Medical Center Göttingen, Göttingen, Germany; 5 Cluster of Excellence “Multiscale Bioimaging: from Molecular Machines to Networks of Excitable Cells” (MBExC), University of Göttingen, Göttingen, Germany; 6 Göttingen Center for Molecular Biosciences (GZMB), Research Group Structure and Function of Molecular Machines, University of Göttingen, Göttingen, Germany

**Keywords:** Cryoelectron microscopy, RNA, Viral infection, Transcription

Correction to: *Nature Communications* 10.1038/s41467-025-57219-5, published online 06 March 2025

In this article reference 21 was incorrect and should have been ‘Balıkçı, E., Günl, F., Carrique, L., Keown, J. R., Fodor, E., & Grimes, J. M. (2025). Structure of the Nipah virus polymerase complex. The EMBO Journal, 44(2), 563-586’. The original article has been corrected.

