# Supplementary material for: Structural basis of Nipah virus RNA synthesis
Source: Nat Commun. 2025 Mar 6;16:2261. doi: 10.1038/s41467-025-57219-5 (PMC11885841; doi:10.1038/s41467-025-57219-5)
Supplement: Supplementary file 1 — Supplementary Information [file 41467_2025_57219_MOESM1_ESM.pdf]

SUPPLEMENTARY INFORMATION

Structural basis of Nipah virus RNA synthesis

Fernanda A. Sala[1,2], Katja Ditter[1,2], Olexandr Dybkov[3], Henning Urlaub[3,4,5] and Hauke S. Hillen[1,2,5,6*]

[1]Department of Cellular Biochemistry, University Medical Center Göttingen, Göttingen, Germany

[2]Research Group Structure and Function of Molecular Machines, Max Planck Institute for Multidisciplinary Sciences, Göttingen, Germany

[3]Bioanalytical Mass Spectrometry Group, Max Planck Institute for Multidisciplinary Sciences, Göttingen, Germany

[4]Bioanalytics Group, University Medical Center Göttingen, Institute for Clinical Chemistry, Göttingen, Germany

[5]Cluster of Excellence "Multiscale Bioimaging: from Molecular Machines to Networks of Excitable Cells" (MBExC), University of Göttingen, D-37075 Göttingen, Germany

[6]Göttingen Center for Molecular Biosciences (GZMB), Research Group Structure and Function of Molecular Machines, University of Göttingen, D-37077 Göttingen, Germany

*Corresponding author. E-mail: hauke.hillen@med.uni-goettingen.de

This PDF file includes:

Figures S1 to S8

Table S1

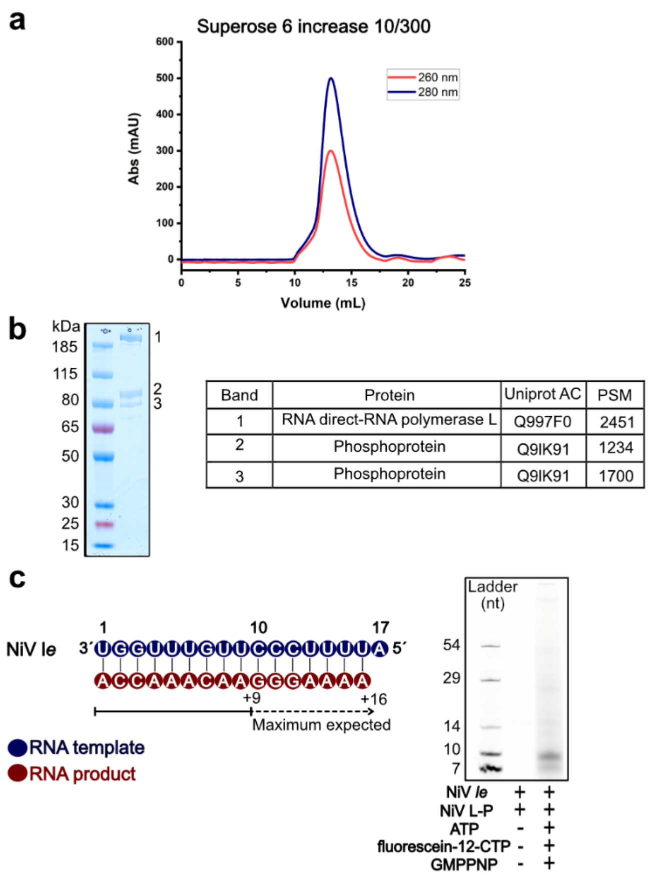

Band	Protein	Uniprot AC	PSM
1	RNA direct-RNA polymerase L	Q997F0	2451
2	Phosphoprotein	Q9IK91	1234
3	Phosphoprotein	Q9IK91	1700

Supplementary Fig. 1: Purification and activity of recombinant NiV L-P complex.

a) Size exclusion chromatography profile of the NiV L-P complex using a Superose 6 Increase 10/300 GL column. The absorbance at 260 nm and 280 nm is shown in red and blue, respectively. Source data are provided as a Source Data file.

b) SDS-Page analysis of the purified NiV L-P complex using a 4-12% polyacrylamide gel. Proteins in visible bands were identified by mass spectrometry. The top hit for each band and respective number of peptide-spectrum matches (PSM) are shown in a table. Due to a lower intensity of band 3, 3 times more of this sample was injected into the mass spectrometer as compared to the bands 1 and 2.

c) RNA synthesis assay using fluorescein-labeled CTP. Products were separated on a 20% urea gel and visualized using with a Typhoon phosphorimager (GE Healthcare,). RNA product lengths were determined by comparing them to a ladder of synthetic RNA molecules of known sizes. The experiment was performed in triplicate. Source data are provided as a Source Data file.

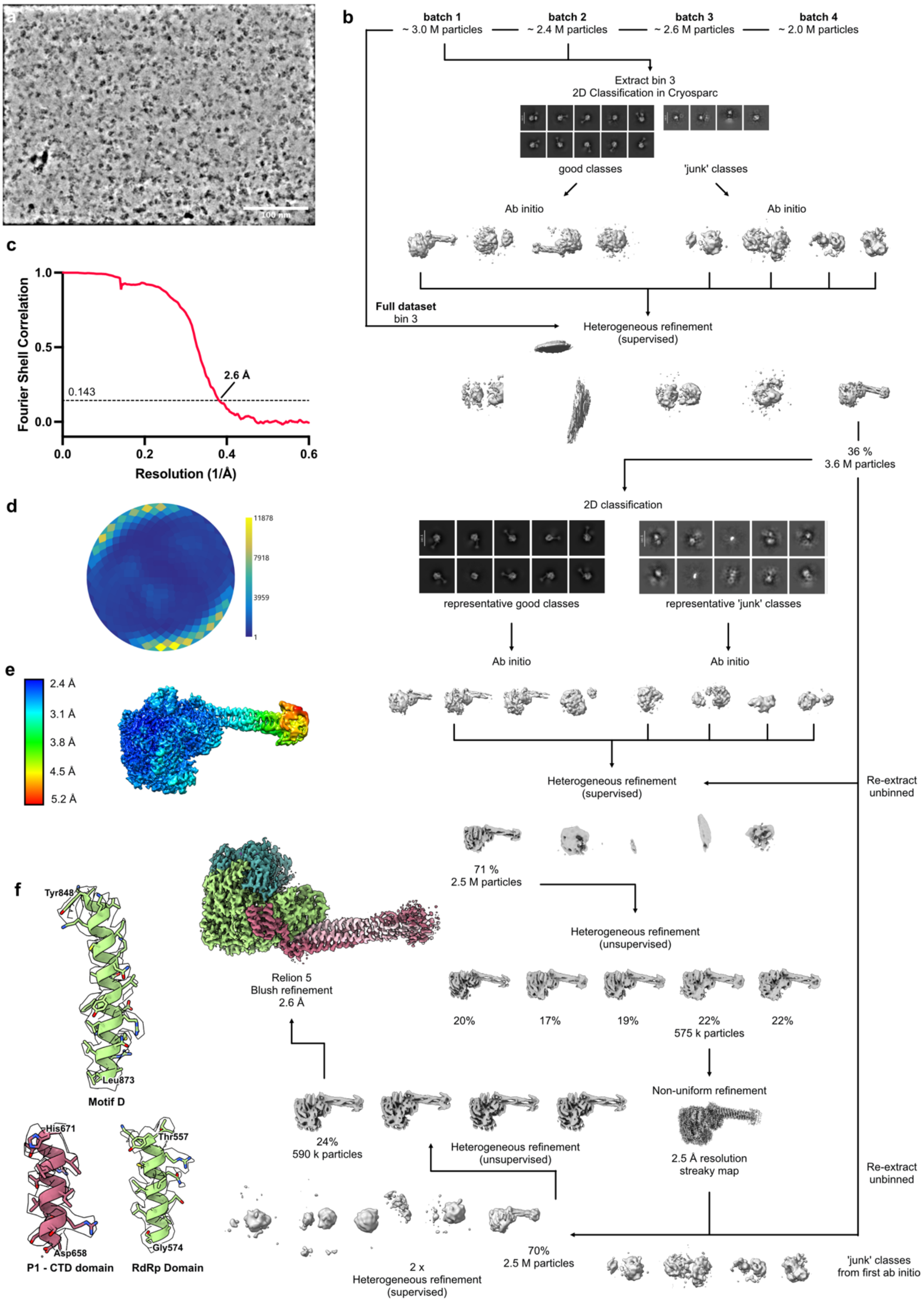

Supplementary Fig. 2: Cryo-EM processing workflow for the apo NiV L-P complex.

a) Representative denoised micrograph.

b) Schematic of image processing workflow. See Methods for details.

c) FSC plot of final reconstruction.

d) Angular distribution plot created with Warp[48].

e) Local resolution filtered map (created in Relion 5.0[50]) colored by local resolution.

f) Densities and atomic models for representative structural elements.

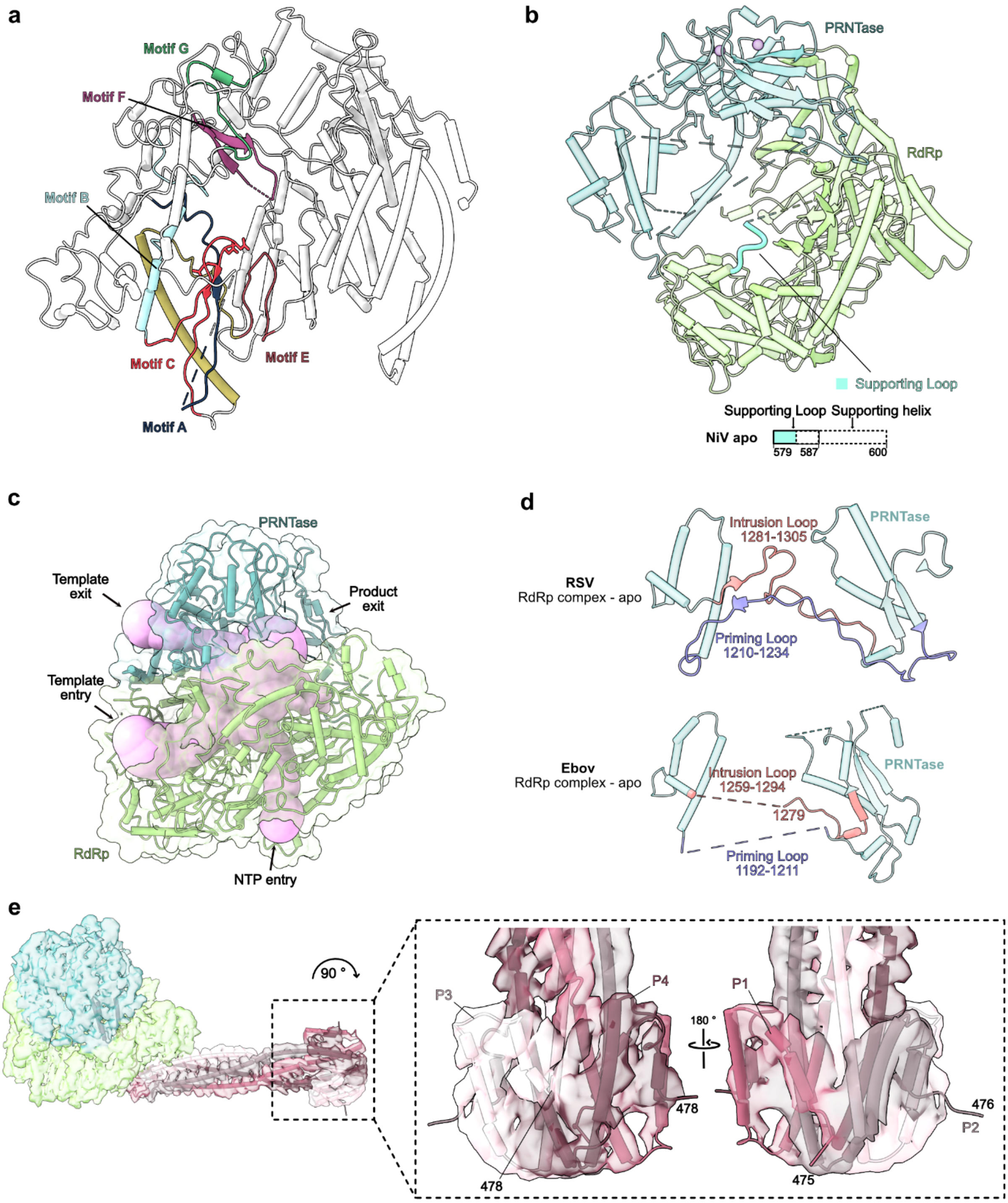

Supplementary Fig. 3: Details and comparison of apo NiV L-P complex.

a) Cartoon depiction of conserved catalytic motifs. The RdRp domain is color-coded with catalytic motifs from A to G displayed and labeled in distinct colors.

b) RdRp and PRNTase domains are shown as cartoon with structural and schematic depiction of the supporting helix and loop.

c) Presumable RNA tunnels within the NiV L-P complex. Cavities in the complex were calculated using CAVERweb[60]Click or tap here to enter text. and are represented by pink spheres.

d) Comparison of the priming and intrusions loops of in apo NiV L-P complex with the apo RSV L-P complex (PDB ID: 6UEN)[27] and apo EBOV L-P complex (PDB ID: 7YER)[10]. e) Detailed view of the unique P-stalk tip of the NiV L-P complex. The apo NiV L-P complex structure is displayed as cartoon with the local resolution filtered cryo-EM map as transparent surface.

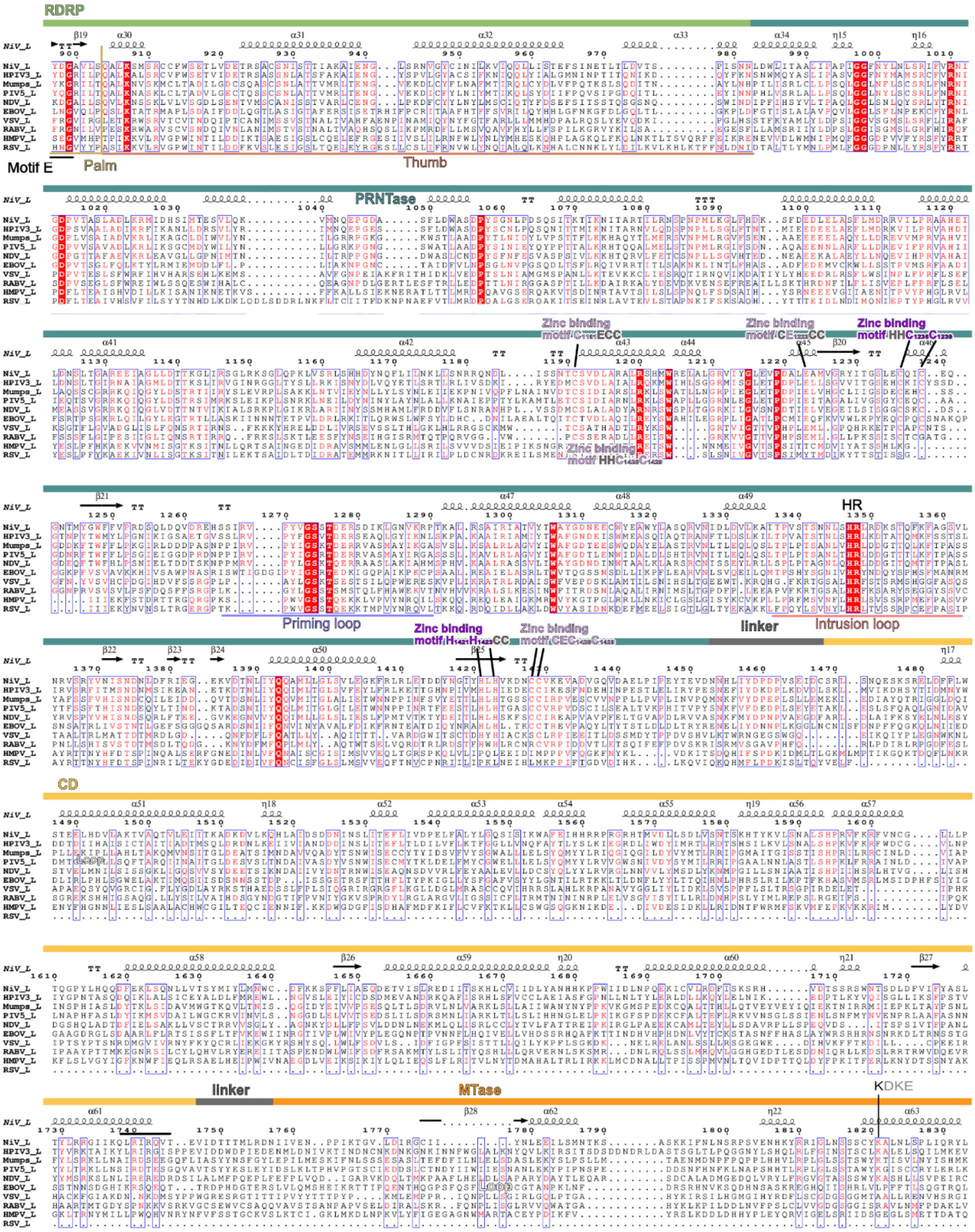

RDRP
Motif E
Palm
Thumb
PRNTase
Zinc binding
Priming loop
linker
Intrusion loop
HR
CD
linker
MTase
KDKE
NiV_L
HPIV3_L
Mumps_L
PIV5_L
NDV_L
EBOV_L
VSV_L
RABV_L
HMPV_L
RSV_L

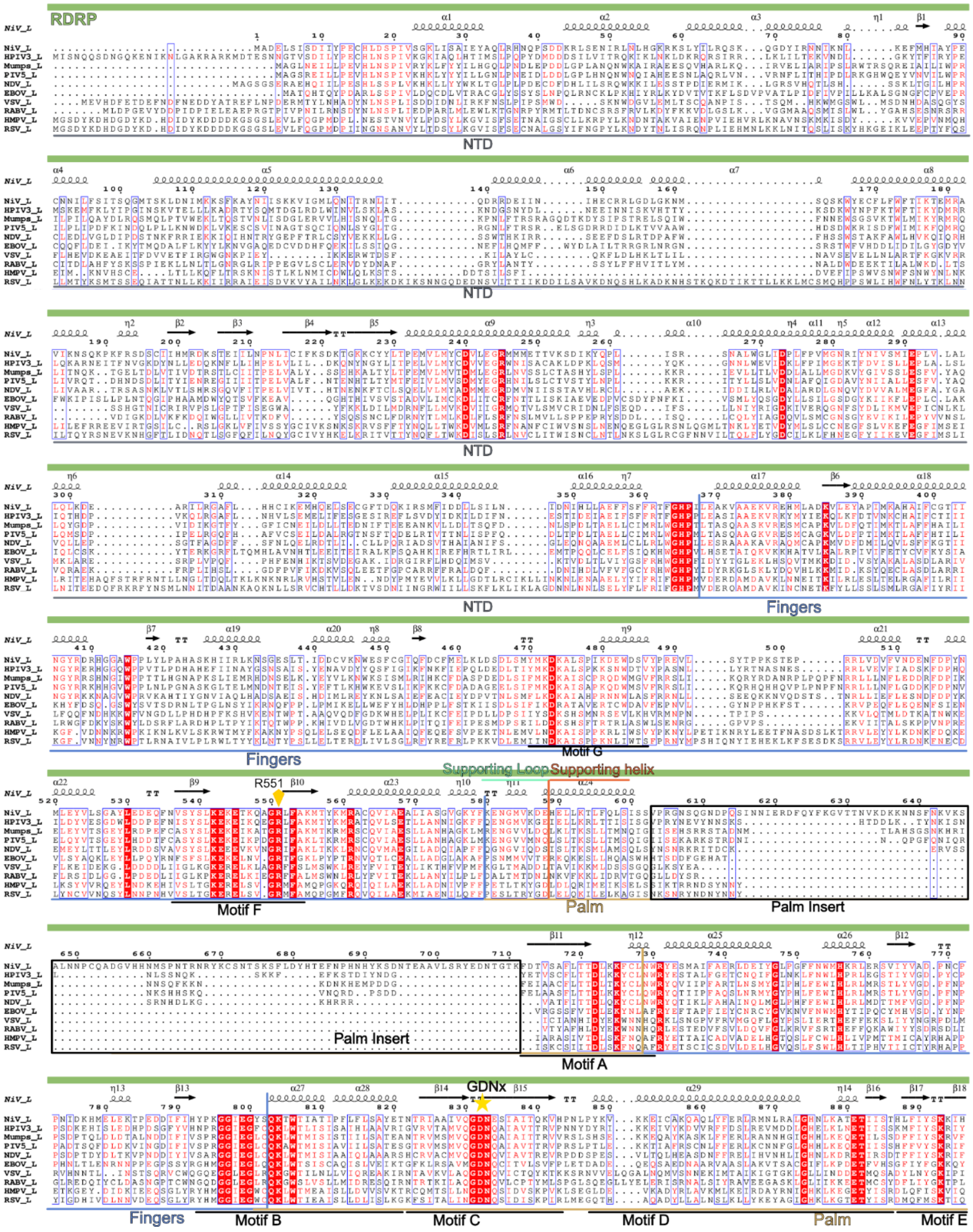

RDRP
NTD
Fingers
Motif G
Supporting Loop
Supporting helix
R551
Motif F
Palm
Palm Insert
Motif A
GDNx
Motif B
Motif C
Motif D
Motif E

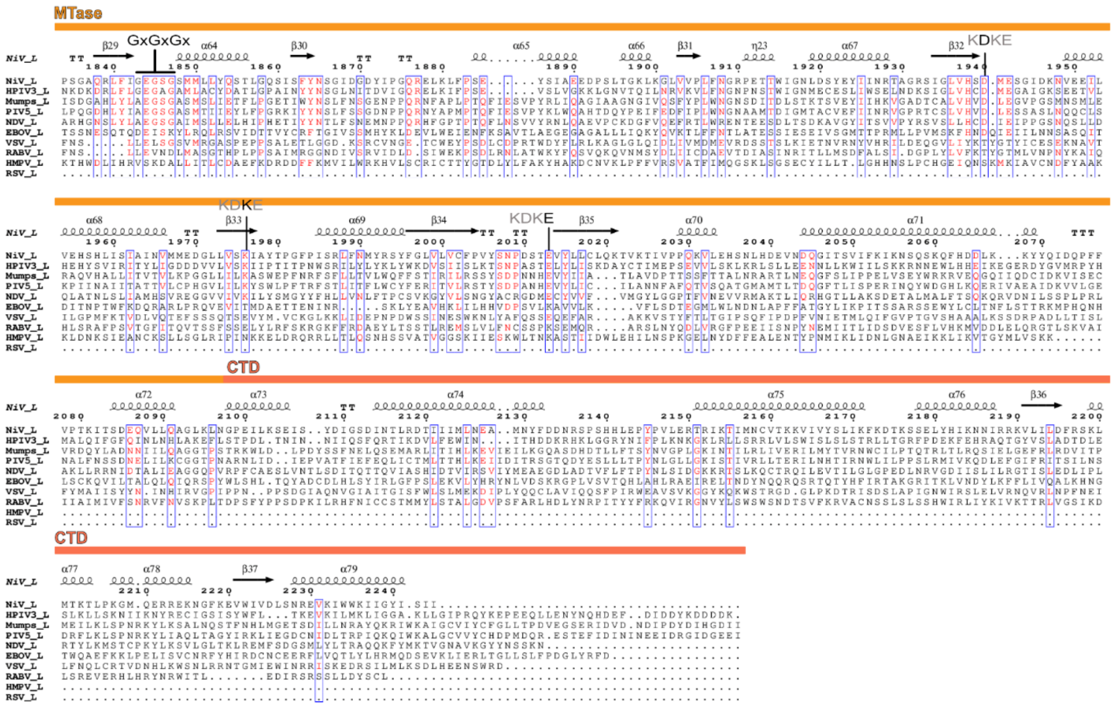

Supplementary Fig. 4: Structure-based sequence alignment of nsNSV L proteins. Sequences for Nipah virus, hPIV3, Mumps virus, PIV5, NDV, EBOV, VSV, RABV, HMPV, and RSV. Multiple sequence alignments were performed with MultAlin[61] and visualized with ESPript3[62]. Secondary structures, domain boundaries, conserved regions, and key motifs are indicated.

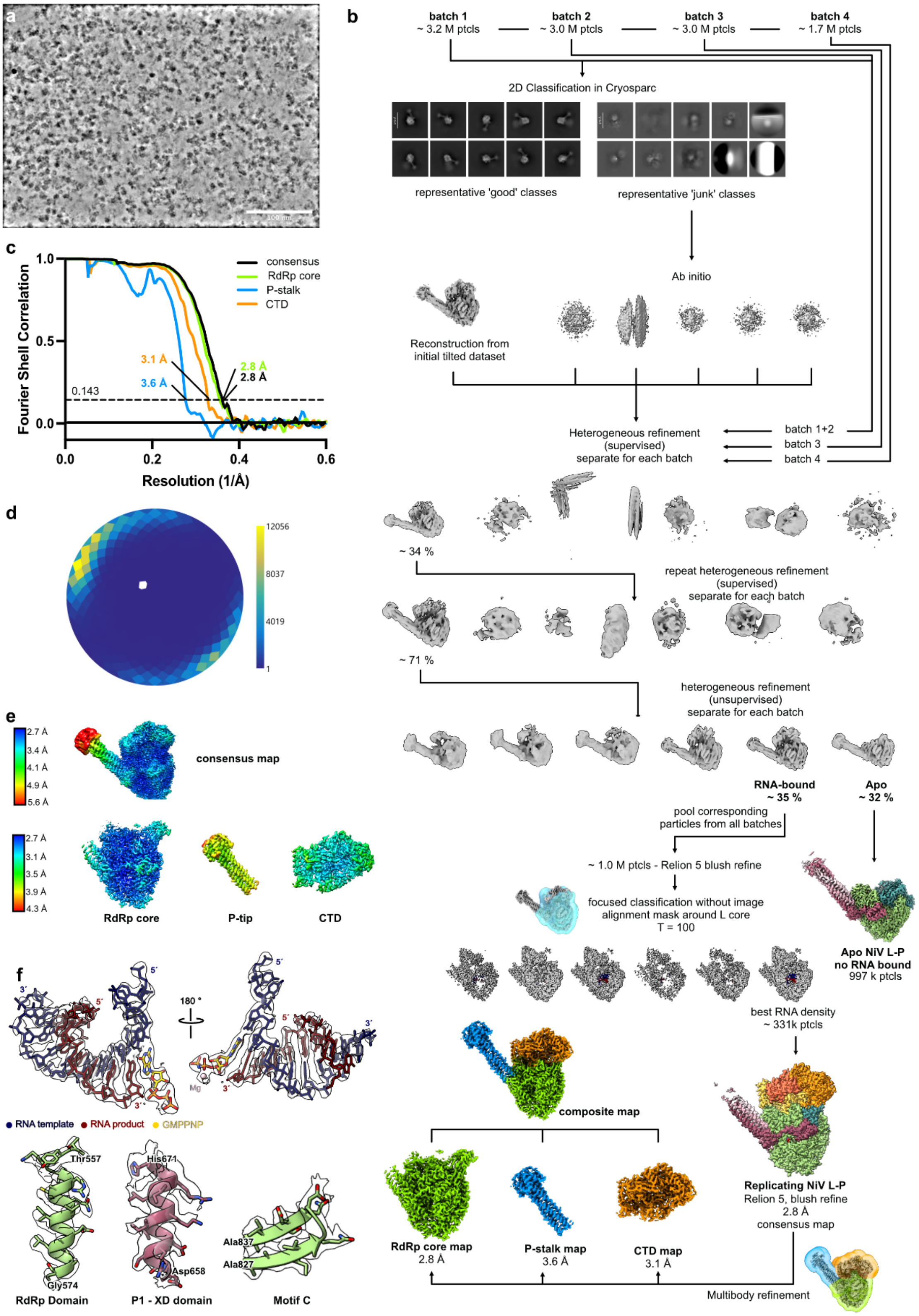

Supplementary Fig. 5: Cryo-EM processing workflow for the elongating NiV L-P complex.

a) Representative denoised micrograph.

b) Schematic of image processing workflow. See Methods for details.

c) FSC plot of final reconstruction.

d) Angular distribution plot created with Warp[48].

e) Local resolution filtered maps (created in Relion 5.0[50]) colored by local resolution.

f) Composite cryo-EM density map and atomic models for ligands and representative structural elements.

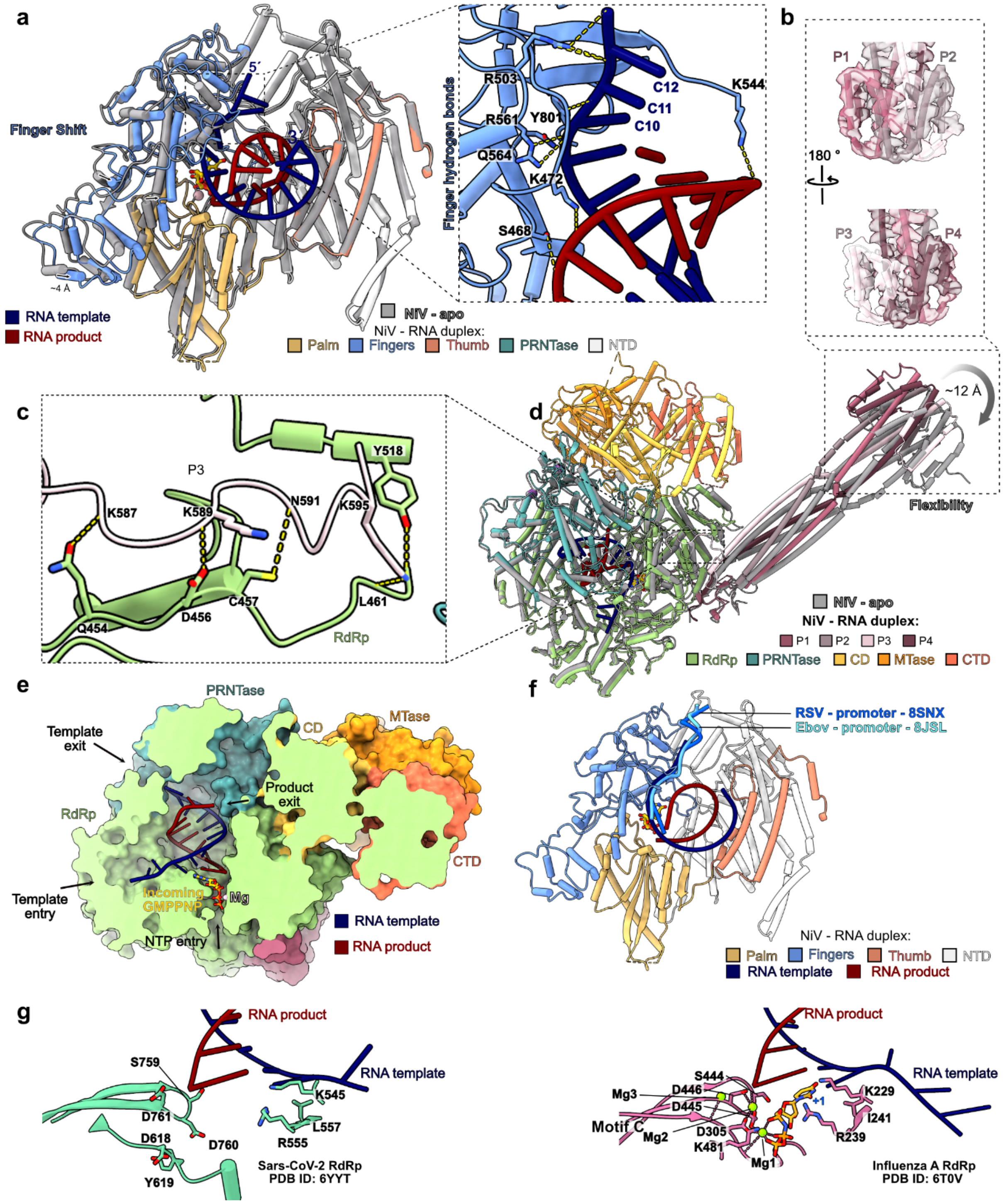

Supplementary Fig. 6: Details and comparison of elongating NiV L-P complex.

a) Superimposition of the NiV L RdRp domain in apo (gray) and elongating state (color-coded: finger domain in blue, thumb in salmon, palm in golden yellow, and NTD in gray). The active site and GMPPNP are shown as sticks, with Mg^{2+} ions as pink spheres. The inset displays the interactions between the finger domain and the RNA template, with hydrogen bonds indicated by dashed lines.

b) Close-up view of the P-stalk tip of elongating NiV L-P complex. The apo NiV L-P complex structure is displayed as cartoon with the composite cryo-EM map as transparent surface.
c) Close-up view of the interactions between P3 and the L RdRp domain.
d) Superimposition of apo (gray) and elongating (colored) NiV L-P complex structures shows conformational flexibility of the P-stalk.
e) Slice-through view of elongating NiV L-P complex. The structure is displayed as surface representation with a partially clipped surface. The RNA template and GMPPNP are shown as cartoons and stick, respectively. The template entrance and exit, the NTP entrance and product exit are highlighted
f) Comparison to promoter-bound nsNSV L-P structures. The RdRp domain of elongating NiV L is colored as in panel a. The structures of EBOV (PDB ID: 8JSL)[16] and RSV L-P complex (PDB ID: 8SNX)[17] in complex with promoter RNA were superimposed and the RNA is shown as ribbon. The template RNA follows a similar trajectory in all complexes.
g) Conserved active site architecture across different RNA virus polymerases. (Left) Close-up view of the SARS-CoV-2 RdRp active site (PDB ID: 6YYT)[34]. (Right) Close-up view of the Bat-Influenza A RdRp active site (PDB ID: 6T0V)[35]. Residues interacting with RNA are shown as sticks. The incoming NTP is depicted as yellow sticks and Mg^{2+} ion as green spheres.

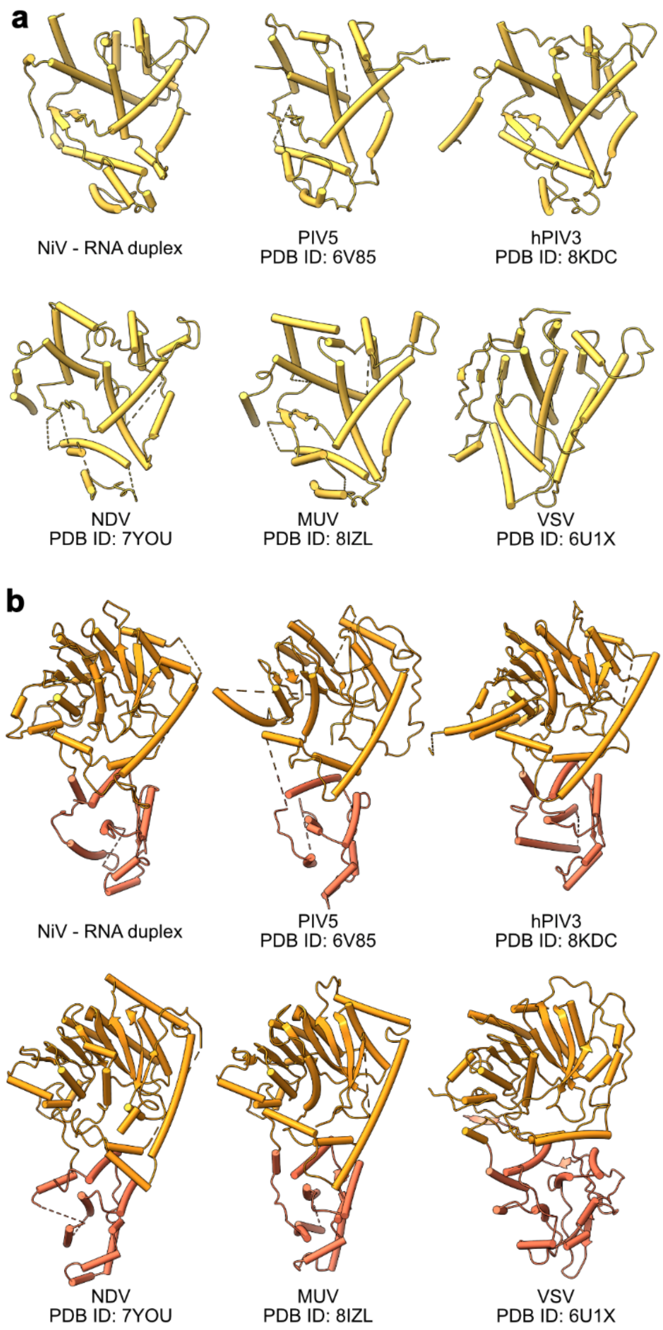

Supplementary Fig. 7: Structural comparison of CD and MTase from NiV and related viruses.

a) Comparison of the CD domains of Nipah virus, parainfluenza virus 5 (PIV5, PDB ID: 6V85)[14], human parainfluenza virus 3 (hPIV3, PDB ID: 8KDC)[13], Newcastle disease virus (NDV, PDB ID: 7YOU)[18], Mumps virus (PDB ID: 8IZL)[15], and vesicular stomatitis virus (VSV, PDB ID: 6U1X)[19].

b) Comparison of the methyltransferase (MTase) domain (colored in gold yellow) and CTD (orange) of the viruses described in (a)

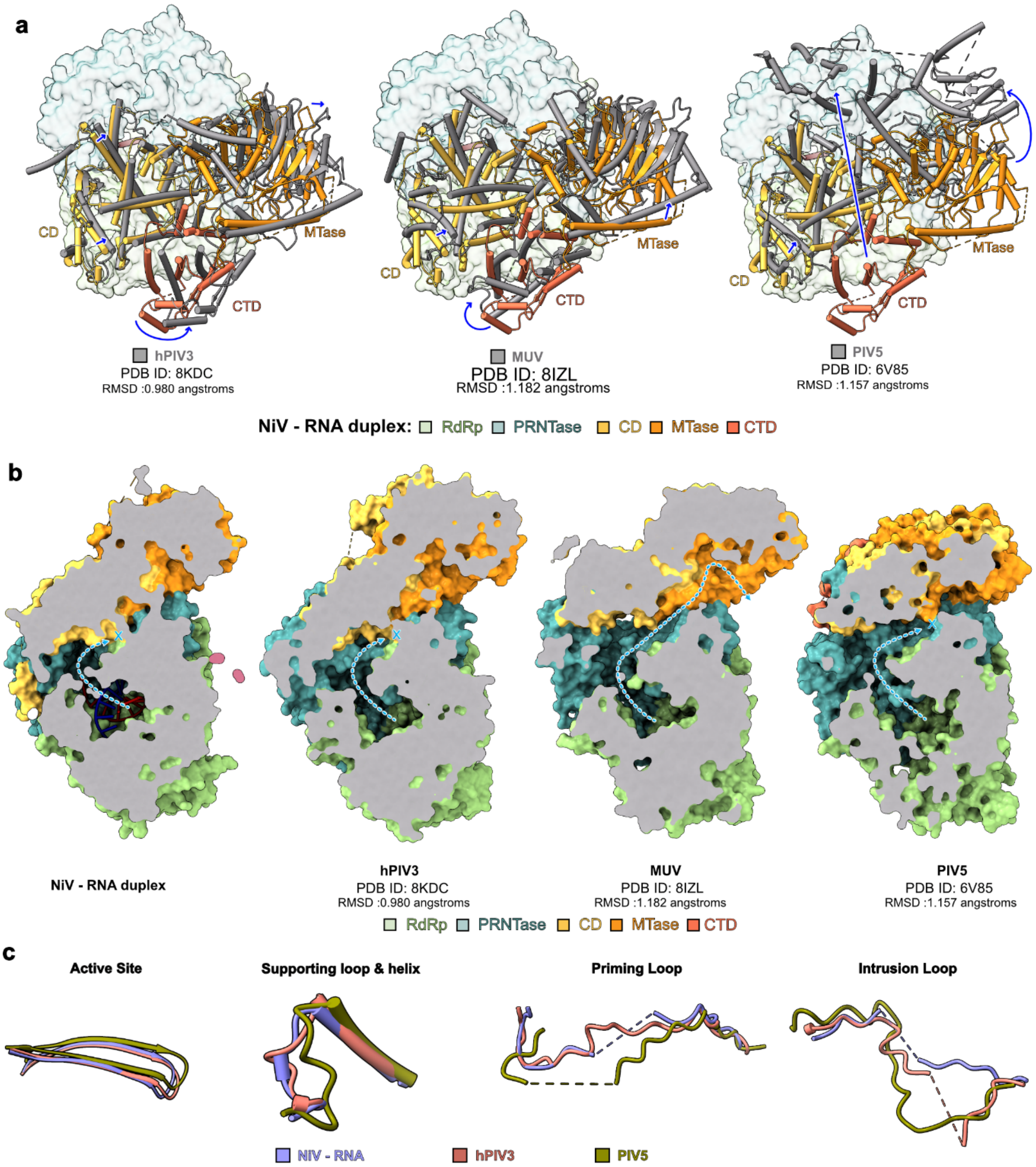

Supplementary Fig. 8: Structural comparison of L flexible domains from NiV and related viruses.

a) Superimposition of the L RdRp domain of elongating NiV L-P complex with hPIV3 (left, PDB ID: 8KDC, RMSD: 0.980 Å)[13], MUV (middle, PDB ID: 8IZL, RMSD: 1.182 Å)[15] and PIV5 (right, PDB ID: 6V85, RMSD: 1.157 Å)[14]. The RdRp and PRNTase domains are displayed as surfaces in light green and dark cyan, respectively. The CD, MTase, and CTD regions are shown as cartoons, colored as in Fig. 2a for elongating NiV L-P complex and in gray for the other structures.

b) Slice-through views of elongating L-P complexes of nsNSVs, aligned as in panel a). The structures are shown in surface representation with a partially clipped surface, highlighting the RNA product exit tunnel.

c) Comparison of active site motif C, supporting loop amd helix, priming loop, and intrusion loop between NiV, hPIV3 (PDB ID: 8KDC)[13], and PIV5 (PDB ID: 6V85)[14] L-P complexes. Color-coding as indicated.

SUPPLEMENTARY TABLE

Supplementary Table 1 | Cryo-EM data collection, refinement and validation statistics

	**Apo Nipah L-P complex**<br>**PDB: 9GJT**<br>**EMD-51402**	**Elongating Nipah L-P complex**<br>**PDB: 9GJU**<br>**EMD-51403**
**Data collection and processing**		
Magnification	105,000 x	105,000 x
Voltage (kV)	300	300
Electron exposure (e–/Å^2)	48.94	52.00
Defocus range (μm)	0.5 – 2.5	0.5 – 2.5
Pixel size (Å)	0.834	0.834
Symmetry imposed	C1	C1
Initial particle images (no.)	9,886,170	10,957,464
Final particle images (no.)	591,312	330,750
Map resolution (Å)	2.6	2.8
FSC threshold	0.143	0.143
Map resolution range (Å)	2.4 – 5.2	2.7 – 5.6
Map sharpening *B* factor (Å^2)	-93	-47
**Refinement**		
Model resolution (Å)	2.7	2.8
FSC threshold	0.5	0.5
Model composition		
Non-hydrogen atoms	14,017	20,948
Protein residues	1,741	2,540
Ligands	2 x ZN	2 x ZN, 1 x MG, 1 x GMPPNP
B factors (Å^2)		
Protein	81.5	54.6
Ligand	96.8	21.7
R.m.s. deviations		
Bond lengths (Å)	0.003	0.002
Bond angles (°)	0.541	0.481
Validation		
MolProbity score	1.19	1.32
Clashscore	3.99	5.74
Poor rotamers (%)	0.44	1.03
Ramachandran plot		
Favored (%)	98.02	98.41
Allowed (%)	1.92	1.48
Disallowed (%)	0.06	0.12
